# Development of a Cracking Resistance Index for Ballastless Track Slab Concrete: Evaluation and Mixture Optimization

**DOI:** 10.3390/ma19194192

**Published:** 2026-09-30

**Authors:** Li Yang, Hailin Lu, Jing Hao, Jicheng Zhang, Zhigang Zhu, Yanping Tu

**Affiliations:** 1 China Railway 15th Bureau Group Co., Ltd., Shanghai 200070, China; 13247187871@163.com; 2 School of Smart City and Life Health, Wuchang Shouyi University, Wuhan 430064, China; luhailin@wit.edu.cn; 3 School of Civil Engineering and Architecture, Wuhan Institute of Technology, Wuhan 430074, China; 04049006@wit.edu.cn (Z.Z.); 04001072@wit.edu.cn (Y.T.); 4 School of Urban Construction and Safety Engineering, Hubei University of Education, Wuhan 430064, China; hh94959@126.com

**Keywords:** ballastless track slab, cracking resistance index, mixture optimization, response surface methodology, internal curing, shrinkage deformation

## Abstract

Ballastless track slabs are subjected to complex restraint conditions and operational environments, making deformation-induced cracking a critical factor affecting durability and service performance. Conventional cracking resistance evaluations generally rely on individual parameters, which cannot adequately describe the relationship between cracking driving forces and resistance capacity under restrained conditions. This study proposes a cracking resistance evaluation and mixture optimization framework for ballastless track slabs. A cracking resistance index (CRI) was developed based on the competition between deformation-induced stress demand and flexural cracking resistance by integrating shrinkage deformation, elastic modulus, and flexural strength. The proposed CRI is intended as a comparative index for quantitatively assessing the relative cracking resistance among different concrete mixtures. The effects of key mixture parameters, including water-to-binder ratio, sand ratio, binder content, air-entraining agent dosage, and internal curing admixture dosage, on fresh properties, mechanical performance, shrinkage behavior, and CRI were systematically investigated. The results indicated that the water-to-binder ratio, air-entraining agent dosage, and internal curing admixture dosage were the dominant factors affecting CRI. In particular, internal curing reduced shrinkage deformation and consequently decreased the CRI, which may be attributed to improved internal moisture conditions. Based on the Box–Behnken design method, a response surface model (RSM) was established to optimize mixture proportions with the objective of minimizing CRI while satisfying the mechanical requirements for C40 ballastless track slab concrete. A preliminary experimental verification using three replicate specimens prepared with the optimized mixture showed good consistency between the RSM-predicted and measured CRI values, with a relative error of 3.81%. The proposed framework provides a quantitative approach for cracking resistance-oriented mixture optimization of concrete used in ballastless track slab.

## 1. Introduction

Ballastless track systems have become an essential component of modern high-speed railway infrastructure due to their advantages in structural stability, operational safety, and reduced maintenance requirements [1]. As a key load-bearing element of high-speed railway systems, ballastless track slabs must maintain sufficient mechanical performance and durability throughout their service life. However, these concrete components are continuously exposed to complex environmental and mechanical conditions, including temperature fluctuations, shrinkage deformation, deformation restraint, and repeated dynamic loading caused by train operations [2,3]. Among these deterioration mechanisms, deformation-induced cracking has been recognized as one of the most critical factors affecting the long-term durability and service reliability of ballastless track structures [4,5,6,7,8].

Compared with conventional concrete members, ballastless track slabs exhibit distinct structural characteristics, including large dimensions, high stiffness, and strong interactions with supporting layers and adjacent components. These characteristics significantly restrict the free deformation of concrete caused by temperature variation and moisture loss. As a result, the imposed deformation cannot be completely released and is partially converted into internal tensile stress. When the accumulated tensile stress approaches the cracking resistance of concrete, microcracks may initiate and gradually develop into visible cracks under sustained environmental exposure and cyclic loading [9,10]. Therefore, the cracking behavior of ballastless track slab concrete is fundamentally controlled by the interaction between deformation-induced stress development and the inherent cracking resistance of the material.

In recent years, considerable efforts have been devoted to improving the cracking resistance of concrete through material optimization and mixture design strategies. Various approaches, including optimization of binder systems, modification of aggregate structures, and incorporation of functional admixtures, have been proposed to reduce shrinkage deformation and enhance durability [11,12,13,14,15]. Among these approaches, internal curing has attracted increasing attention because it can provide additional internal moisture during hydration, reduce self-desiccation, and mitigate shrinkage-induced deformation [16,17,18]. Parallel to these material-centered approaches, statistical mixture design methodologies have gained traction as a means of striking a balanced trade-off among multiple performance attributes. Conventional assessment methods generally rely on individual parameters, such as shrinkage deformation, elastic modulus, tensile strength, or flexural strength. Although these parameters provide important insights into deformation behavior or mechanical resistance, they mainly characterize isolated aspects of cracking mechanisms and cannot adequately reflect the interaction between deformation-induced cracking tendency and resistance capacity [19,20]. A mixture with lower shrinkage deformation may generate reduced stress under restrained conditions; however, insufficient mechanical resistance may still result in poor cracking resistance. Conversely, increasing concrete strength does not necessarily lead to improved cracking resistance, as a higher elastic modulus may increase stress development during restrained deformation. Therefore, evaluating cracking resistance solely based on individual parameters may not provide a comprehensive understanding of the factors governing cracking behavior of ballastless track slab concrete.

The limitations of conventional evaluation methods are particularly significant for ballastless track slab concrete. In practical service conditions, cracking rarely occurs under a simple uniaxial tensile stress state [21]. Instead, temperature gradients, nonuniform shrinkage, slab deformation, reinforcement restraint, and interface constraints collectively produce complex stress distributions within the concrete slab. Under such conditions, bending-induced tensile stress may become a dominant factor controlling crack initiation. Therefore, mechanical parameters capable of representing bending resistance, such as flexural strength, may provide a more suitable characterization of cracking resistance for ballastless track slab concrete than direct tensile strength [22,23,24].

To address this issue, this study proposes a cracking resistance index (CRI) based on the fundamental competition between deformation-induced stress demand and flexural cracking resistance. Unlike conventional single-parameter indicators, the proposed index incorporates shrinkage deformation, elastic modulus, and flexural strength into a unified framework, thereby providing a quantitative description of the balance between crack initiation tendency and material resistance. The effectiveness of CRI was investigated through systematic analysis of key mixture parameters, including water-to-binder ratio, sand ratio, binder content, air-entraining agent dosage, and internal curing agent dosage. Furthermore, response surface methodology was employed to optimize the concrete mixture with the objective of minimizing CRI while satisfying the mechanical requirements of C40 ballastless track slab concrete. The objectives of this study are therefore to: (1) establish CRI for evaluating the cracking resistance of concrete; and (2) develop an optimized mixture design that improves cracking resistance. It should be emphasized that the proposed CRI is a comparative evaluation index characterizing the competition between deformation-induced stress demand and flexural resistance capacity, rather than a comprehensive fracture mechanics model incorporating fracture-related parameters or cohesive crack model parameters.

The remainder of this paper is organized as follows. Section 2 introduces the development and application of the CRI. First, the crack initiation mechanism of concrete ballastless track slabs is analyzed to establish the theoretical basis of the proposed index. Subsequently, the physical interpretation and calculation framework of CRI are presented, followed by its application in evaluating the cracking resistance of concrete mixtures. Section 3 describes the materials, experimental methods, and experimental design, and discusses the effects of mixture parameters on the mechanical properties and cracking resistance of concrete. Section 4 develops response surface models based on CRI and investigates the effects of mixture variables on the proposed index. The optimal mixture proportion is determined and experimentally verified. Finally, the main findings and conclusions of this study are summarized in Section 5.

## 2. Development and Application of CRI

### 2.1. Crack Initiation Mechanism of Concrete Ballastless Track Slabs

During service, ballastless track slab concrete is subjected to the combined effects of environmental and mechanical actions, including temperature variations, shrinkage deformation, structural restraint, and cyclic train loading. Among these factors, cracking induced by shrinkage deformation is one of the primary mechanisms governing the durability of concrete structures; thus, the present study focuses on shrinkage-induced deformation and restraint effects.

A classical relationship, derived from the basic mechanics of restrained deformation and widely adopted in previous studies to evaluate tensile stress, can be expressed as:

(1) *σ*_res_ = *E*_c_*ε*_res_*η*

where *σ*_res_ represents the shrinkage-induced tensile stress; *E*_c_ denotes the elastic modulus of concrete; *ε*_res_ represents the shrinkage strain; and *η* is the degree of restraint, which characterizes the extent to which free deformation is converted into tensile stress.

The restraint degree is influenced by various factors, including structural configuration, reinforcement ratio, interface conditions, geometric constraints, and environmental exposure conditions. A higher restraint degree results in a greater conversion of free deformation into tensile stress, thereby increasing the cracking potential of concrete. Equation (1) therefore indicates that the cracking tendency of restrained concrete is governed not only by the magnitude of free deformation but also by the elastic stiffness of the material and the degree of structural restraint.

Crack initiation occurs when the tensile stress generated by restrained deformation exceeds the available resistance of concrete. Therefore, the cracking process can be fundamentally described as a competition between the deformation-induced stress demand and the material resistance capacity. This relationship can be expressed as:

(2) *E*_c_*ε*_res_*η* ≥ *f*_d_

where *f*_d_ represents the tensile resistance of concrete.

Likewise, the comparative cracking resistance of concrete can be evaluated by the ratio between the stress demand induced by restrained deformation and the corresponding resistance capacity. Based on this concept, Ref. [25] introduced the following anti-cracking coefficient to characterize the cracking tendency of restrained concrete:

(3) $$K\;=\;\frac{E_{c}\varepsilon_{\mathrm{res}}\eta }{f_{d}}$$

where *K* is the anti-cracking coefficient. A higher value of *K* indicates that the stress demand is closer to the available cracking resistance and, consequently, a higher susceptibility to crack initiation.

Although Equation (3) provides a rational basis for evaluating the cracking tendency of restrained concrete, its direct application to ballastless track slabs requires further consideration of the actual stress state and cracking characteristics of the slab structure.

### 2.2. Development and Physical Interpretation of CRI

The cracking behavior of ballastless track slabs is not governed exclusively by a uniform uniaxial tensile stress state. In practical track structures, temperature gradients and nonuniform shrinkage can generate slab warping and bending, while local restraints associated with reinforcement, interfaces, sleepers, structural discontinuities, and geometric variations can further amplify local stress concentrations. As a result, critical regions of a ballastless track slab may experience a bending-dominated tensile stress state rather than pure uniaxial tension. Previous studies have similarly demonstrated that temperature and humidity variations, structural restraint, and slab warping play important roles in crack initiation and propagation in ballastless track structures [7].

For this reason, the direct tensile strength of concrete may not fully represent the cracking resistance of track slab concrete under actual service conditions. Moreover, direct tensile tests can be sensitive to specimen preparation, loading alignment, eccentricity, and local stress concentration, which may introduce considerable experimental variability. In contrast, flexural strength is determined under a stress-gradient condition and directly reflects the ability of concrete to resist crack initiation in the tensile region of a bending member. It therefore provides a more representative mechanical parameter for describing the cracking resistance of ballastless track slab concrete.

This study replaces tensile strength with flexural strength *f*_f_ in Equation (3), so as to capture the bending-dominated stress characteristics of ballastless track slab structures. Accordingly, CRI is proposed to quantitatively evaluate the cracking resistance:

(4) $$K_{\mathrm{CRI}}\;=\;\frac{E_{c}\varepsilon_{\mathrm{res}}\eta }{f_{f}}$$

In laboratory-based cracking resistance evaluation, the restrained condition is conventionally normalized as *η* = 1 to provide a unified reference state for comparing different concrete mixtures. In this study, *η* = 1 is adopted as an idealized and unified reference restraint condition for mixture-level comparative evaluation, rather than as a representation of the actual restraint degree of ballastless track slabs under field conditions. According to Equation (4), different values of *η* would proportionally change the absolute value of CRI, but the comparative capability of CRI for mixture optimization remains unaffected when the same *η* is applied to different mixtures. Therefore, the adoption of *η* = 1 is intended to provide a unified reference condition, under which the relative differences in cracking resistance among different concrete mixtures can be effectively compared. Furthermore, *f*_f_ is not intended to serve as an equivalent substitute for the intrinsic direct tensile strength of concrete, which is more consistent with the bending-dominated cracking behavior considered in this study.

From a mechanical perspective, CRI represents the relative magnitude of the stress demand generated by restrained shrinkage deformation and the available flexural cracking resistance of concrete. A lower CRI value indicates that the concrete possesses a larger resistance margin against deformation-induced cracking, whereas a higher CRI value indicates a smaller margin, implying a less favorable comparative cracking resistance characteristic of the concrete mixture.

Under the adopted mechanical idealization, *K*_CRI_ < 1 indicates that the available flexural cracking resistance exceeds the simplified restraint-induced stress demand, whereas *K*_CRI_ > 1 indicates that the stress demand exceeds the corresponding flexural resistance. However, *K*_CRI_ = 1 should be regarded only as a theoretical reference state rather than as a strict physical threshold for crack initiation. In actual concrete structures, cracking is additionally influenced by creep, stress relaxation, fracture processes, reinforcement effects, local stress concentrations, material heterogeneity, and other nonlinear mechanisms. Therefore, CRI is proposed as an index for evaluating the relative cracking resistance of different concrete mixtures, rather than as a direct predictor of cracking occurrence.

Conventional approaches to evaluating the cracking potential of concrete generally rely on individual material parameters, such as shrinkage deformation, elastic modulus, tensile strength, or flexural strength. Although these parameters are useful for characterizing specific material properties, they cannot independently capture the competing mechanisms governing cracking under restrained conditions. For example, a concrete mixture may exhibit relatively low shrinkage deformation but also possess a low flexural strength. The resulting restraint-induced tensile stress may therefore still approach the cracking resistance of the material. Conversely, another mixture may exhibit greater shrinkage deformation but substantially higher flexural strength, resulting in a greater overall resistance to cracking. Consequently, evaluating cracking resistance solely on the basis of shrinkage deformation or mechanical strength may lead to incomplete or even misleading conclusions.

The proposed CRI was developed based on the previously proposed cracking resistance coefficient and further modified to better represent the stress conditions of ballastless track slab concrete. Compared with single-factor indicators, CRI offers three principal advantages. First, CRI is derived from the fundamental mechanism of restrained cracking rather than from empirical correlations. It directly links the deformation-induced stress development process with the cracking resistance capacity of concrete. Second, CRI simultaneously considers the adverse effects of deformation demand and the beneficial contribution of mechanical resistance. This avoids evaluating shrinkage deformation and strength independently, which may lead to contradictory conclusions. Third, by adopting flexural strength as the resistance parameter, CRI better represents the bending-dominated cracking characteristics of ballastless track slabs.

**Figure 1:**
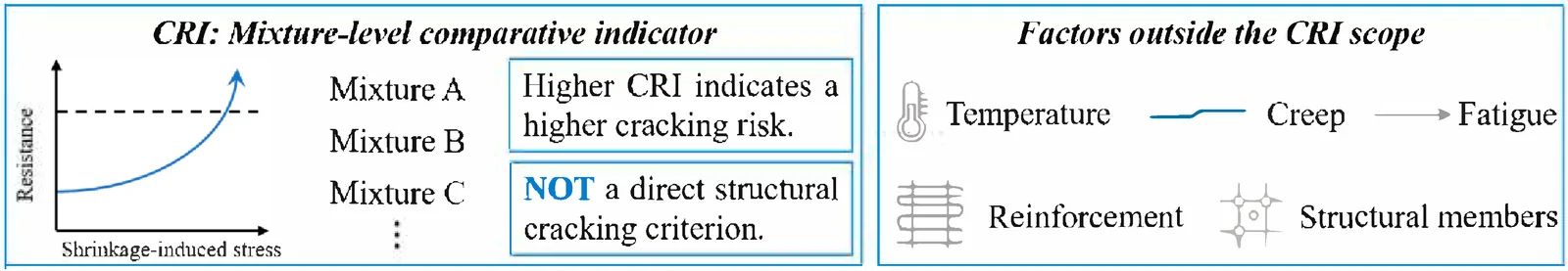
Scope and applicability of the proposed CRI.

Figure 1 illustrates the scope and applicability of the proposed CRI, while highlighting the factors beyond its present scope. Overall, CRI is a comparative indicator at the concrete mixture level, which quantifies the competition between deformation-induced stress demand and flexural cracking resistance under a simplified restraint condition. By integrating these two aspects, CRI provides a meaningful variable for comparing the cracking resistance performance of different concrete mixtures. However, CRI is specifically intended to evaluate shrinkage-related cracking resistance at the mixture level, rather than serving as a direct cracking criterion for structural members.

### 2.3. Application of CRI to Concrete Mixture Optimization

The cracking resistance of ballastless track slab concrete is determined by the combined effects of deformation-induced stress and the cracking resistance of the concrete mixture. Both components are strongly influenced by mixture proportions and may vary in opposite directions. For example, increasing the binder content or reducing the water-to-binder ratio may enhance the mechanical strength of concrete, but may also increase its elastic modulus and shrinkage deformation, thereby increasing the stress generated under restrained conditions. Consequently, optimizing a single material property, such as flexural strength or shrinkage, does not necessarily lead to the optimal overall cracking resistance.

An important advantage of the proposed CRI is that it integrates several material properties directly related to the cracking mechanism of ballastless track slabs. The elastic modulus characterizes the stress response of concrete to restrained deformation, the free deformation represents the deformation demand induced by shrinkage effects, and the flexural strength characterizes the resistance of concrete to bending-induced crack initiation. CRI therefore provides a unified measure of the balance between the cracking driving force and the cracking resistance, rather than assessing these factors separately.

This integrated formulation is particularly advantageous for concrete mixture optimization because changes in mixture proportions can simultaneously affect the *f*_f_, *E*_c_, and *ε*_res_. As a result, an improvement in one individual property may be accompanied by an unfavorable change in another. For instance, a mixture with higher flexural strength does not necessarily possess superior cracking resistance if the corresponding increase in elastic modulus or shrinkage deformation substantially increases the restraint-induced stress demand. By incorporating these competing effects into a single index, CRI can capture the overall influence of mixture proportions on cracking susceptibility.

Accordingly, CRI was selected as the optimization objective in the response surface analysis. The optimization process aims to minimize CRI while maintaining the required mechanical performance of C40 ballastless track slab concrete. The optimization problem can therefore be formulated as:

(5) $$\min\left[\frac{E_{c}(x)\varepsilon_{\mathrm{res}}(x)\eta }{f_{f}(x)}\right]$$

where ***x*** represents the vector of concrete mixture parameters.

In this manner, the proposed optimization strategy does not pursue the improvement of an individual property but instead seeks an optimized balance among deformation characteristics, mechanical performance, and cracking resistance.

## 3. Materials and Experimental Program

### 3.1. Raw Materials

The raw materials used in this study consisted of Portland cement, fly ash, fine aggregate, coarse aggregate, mixing water, and chemical admixtures. Ordinary Portland cement (P·O 42.5) was used as the primary binder. The Class II fly ash with Class F characteristics used as a supplementary cementitious material exhibited a moisture content of 0.3%, a loss on ignition of 3.58%, and a fineness of 18.9%, expressed as the residue retained on a 45 μm square-mesh sieve.

Natural river sand with a fineness modulus of 2.9 was used as fine aggregate, satisfying the requirements for medium sand. Crushed limestone with a nominal particle size range of 5–31.5 mm was adopted as coarse aggregate. Tap water was used as mixing water throughout the experimental program.

**Figure 2:**
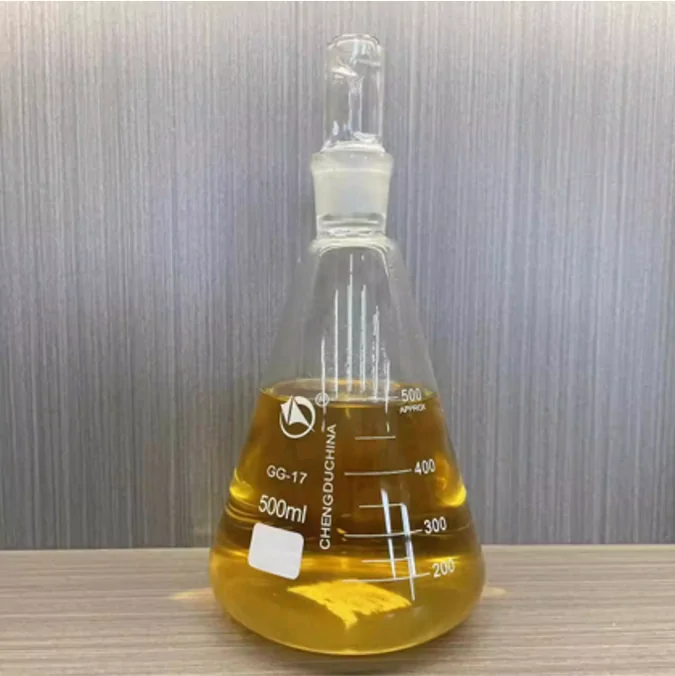
Diluted AH series air-entraining agent.

Three types of chemical admixtures were incorporated, including a polycarboxylate-based high-performance water-reducing agent, an air-entraining agent, and an internal curing admixture. A QX-1 polycarboxylate-based high-performance water-reducing agent with a retarding effect, manufactured by Shanxi Qixuan Building Materials Co., Ltd. (Yuncheng, China), was used. An AH-series air-entraining agent manufactured by Wuhan Jiyesheng Chemical Co., Ltd. (Wuhan, China) was used. The original product was supplied as a pale-yellow powder. To facilitate accurate control of the admixture dosage, the product was diluted with mixing water at a mass ratio of 1:9 and thoroughly mixed to obtain an air-entraining agent with a solid content of 10%, as shown in Figure 2. The internal curing admixture used in this study was a commercial TK-ICM product produced by Hubei Muzhijun Technology Co., Ltd. (Ezhou, China). As listed in Table 1, the corresponding parameters of the internal curing admixture met the specified requirements.

### 3.2. Experimental Methods

#### 3.2.1. Workability Test

The workability of fresh concrete was evaluated by measuring slump and air content according to GB/T 50080-2016 [26]. Slump was measured using a standard Abrams cone. The slump cone was placed vertically and lifted upward at a constant speed, allowing the fresh concrete to deform freely until reaching a stable state. The slump was measured as the vertical difference between the top of the cone and the displaced original center of the top surface of the slumped concrete. The air content of fresh concrete was determined using a pressure-type air meter.

All measurements were conducted immediately after mixing to minimize the influence of time-dependent changes in fresh concrete properties. The workability results were mainly used to evaluate whether the investigated mixture parameters could satisfy the construction requirements of ballastless track slab concrete.

#### 3.2.2. Mechanical Properties Test

The mechanical properties of hardened concrete were characterized by compressive strength, flexural strength, and static elastic modulus tests. All mechanical tests were performed after curing the specimens to the designated ages under standard curing conditions.

The compressive strength was measured using cubic concrete specimens with dimensions of 100 mm × 100 mm × 100 mm. Three specimens were prepared for each concrete mixture. After reaching the specified curing age, the specimens were tested using a TYE-2000B compression testing machine (Wuxi Jianyi Instrument & Machinery Co., Ltd., Wuxi, China). The load was applied continuously and uniformly until specimen failure. The compressive strength was calculated based on the maximum failure load according to the corresponding standard equation. A conversion coefficient of 0.95 was adopted to account for the difference between the measured strength of 100 mm cube specimens and the standard strength value based on 150 mm standard cube specimens, following the GB/T 50081-2019 [27].

The flexural strength was determined using prismatic specimens with dimensions of 100 mm × 100 mm × 400 mm. Three specimens were prepared for each mixture. After curing for the prescribed period, the specimens were subjected to three-point bending tests under continuous loading until failure. The measured flexural strength was adopted as the cracking resistance parameter in the calculation of the CRI.

The static elastic modulus was determined using prismatic specimens of dimensions 150 mm × 150 mm × 300 mm. Compressive loading was applied during the test, and the corresponding deformation was monitored by displacement gauges mounted along the loading direction. The elastic modulus was then calculated from the stress–strain relationship in accordance with GB/T 50081-2019 [27].

#### 3.2.3. Shrinkage Test

Shrinkage deformation is considered one of the major factors contributing to cracking in restrained concrete structures, particularly in ballastless track slab concrete. Therefore, the shrinkage behavior of concrete was investigated using prismatic specimens with dimensions of 100 mm × 100 mm × 515 mm.

During specimen preparation, stainless steel measuring studs were embedded at both ends of each specimen to facilitate accurate length-change measurements. After standard curing for the specified initial period, the specimens were transferred to a controlled drying environment with a temperature of 20 ± 2 °C and a relative humidity of 60 ± 5%. The initial length of each specimen was recorded immediately after exposure to the drying environment. Subsequent length measurements were conducted at predetermined curing ages of 1 d, 3 d, 7 d, 14 d, and 28 d. Here, ‘d’ denotes the curing age in days after casting. The specific procedures followed GB/T 50082-2024 [28].

The shrinkage strain was calculated as follows:

(6) $$\varepsilon_{t}\;=\;\frac{L_{0}\;-\;L_{t}}{L_{b}}$$

where *L*_0_ denotes the initial measuring length (mm), and *L*_t_ represents the measured length change of the specimen (mm).

### 3.3. Experimental Design

Identifying the influence mechanisms of concrete composition parameters and mixture design variables on cracking resistance is of considerable engineering importance. Due to the complexity of concrete composition and the multifaceted nature of cracking mechanisms, extensive studies have been conducted to investigate the effects of material composition, mixture parameters, and environmental conditions on concrete cracking resistance. Nevertheless, these findings remain limited. In particular, existing evaluation methods for the cracking resistance of ballastless track slab concrete are relatively insufficient. This limitation is mainly attributed to the complex nature of concrete cracking, which is governed by the coupled interactions among deformation characteristics, mechanical properties, and internal stress development. Given the multifactorial nature of concrete cracking, selecting appropriate mixture parameters that simultaneously balance deformation characteristics and mechanical resistance is essential for establishing an effective optimization strategy.

The mixtures in this study were designed following the ‘three-low and one-high’ concept, namely a low water-to-binder ratio (W/B), low sand ratio, low total binder content, and high air content. This concept is a qualitative design philosophy that determines the direction in which the mixture proportions are adjusted. To translate this concept into an experimental design, and in combination with internal curing technology, five critical mixture parameters were selected as the independent variables, including W/B, sand ratio, total binder content, air-entraining agent dosage, and internal curing agent dosage. The first three parameters correspond to the ‘three-low’ directions, and the air-entraining agent dosage was selected as the control variable to achieve a relatively high air content, corresponding to the ‘one-high’ direction. The internal curing agent was additionally introduced to reduce shrinkage with the aim of improving cracking resistance.

According to preliminary mixture optimization tests, the W/B was set at 0.33, 0.35, 0.37, and 0.39. The sand ratio was designed as 36%, 39%, 42%, and 45%. The total binder content was selected as 390, 410, 430, and 450 kg/m^3^. The dosage of the AH-series air-entraining agent was determined as 0%, 0.2%, 0.4%, and 0.6% by mass of the total binder content. The TK-ICM internal curing agent was incorporated by the internal mixing method, with dosages of 0%, 3%, 6%, and 9% relative to the total binder mass. The detailed mixture proportions are summarized in Table 2, Table 3, Table 4, Table 5 and Table 6.

The effects of different mixture parameters were evaluated through comprehensive tests on fresh concrete properties, mechanical performance, and shrinkage behavior. Furthermore, the CRI was introduced to quantitatively evaluate the influence of each mixture parameter on comparative cracking resistance under flexural tensile stress conditions. The obtained experimental results provide a fundamental database for subsequent analysis of the effects of mixture parameters on cracking resistance and establish a theoretical basis for the comprehensive evaluation and predictive modeling of concrete cracking resistance using the response surface methodology.

### 3.4. Experimental Results and Discussion

Based on the experimental results, this section discusses the effects of W/B, sand ratio, binder content, air-entraining agent, and internal curing admixture on workability, mechanical properties, and shrinkage behaviors.

**Figure 3:**
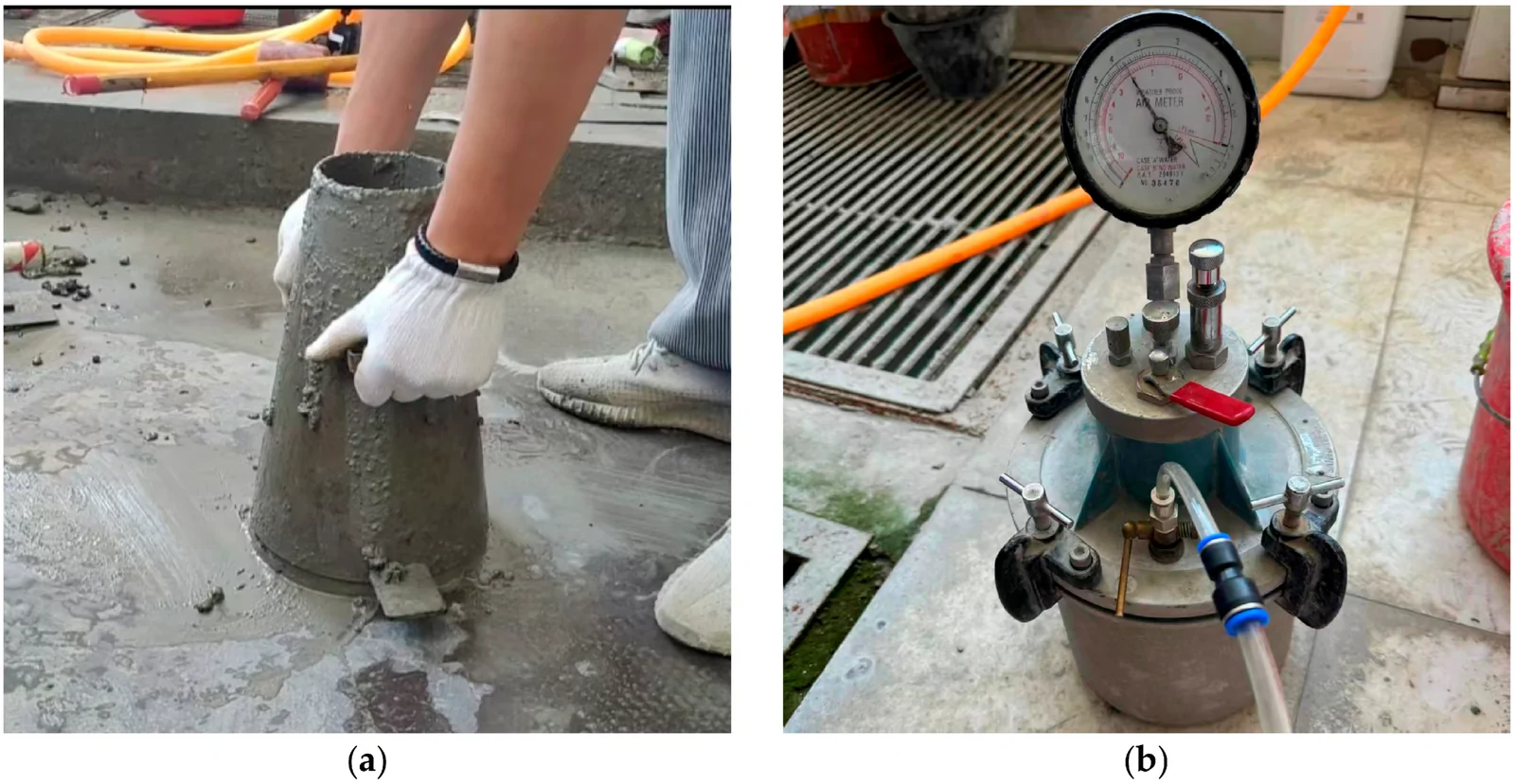
Photographs taken during the test. (**a**) Slump; (**b**) Air content.

The test methods for slump and air content are described in Section 3.2.1, and photographs taken during the slump test and the air content test are presented in Figure 3. The workability results are summarized in Table 7. For each mixture proportion, three parallel specimens were tested, and the average values were adopted as the representative experimental results to minimize the influence of specimen-to-specimen variability. The coefficient of variation among the three specimens was maintained below 15% for all mixtures, ensuring acceptable consistency and repeatability of the experimental data. All concrete mixtures exhibited satisfactory flowability, with slump values ranging from 150 to 210 mm, indicating that the designed mixtures met the basic construction requirements for ballastless track slab concrete. The investigated mixture parameters influenced fresh concrete properties through different mechanisms. Increasing W/B and binder content improved flowability by increasing the amount of free water and paste volume, respectively. In contrast, higher sand ratio and internal curing admixture dosage reduced slump due to increased surface area and additional water demand. The air-entraining agent improved workability by introducing additional microscopic air bubbles, which reduced internal friction between solid particles. Overall, the variations in fresh properties remained within an acceptable range, suggesting that the selected mixture parameters mainly affected hardened properties and cracking resistance rather than causing unacceptable workability deterioration.

**Figure 4:**
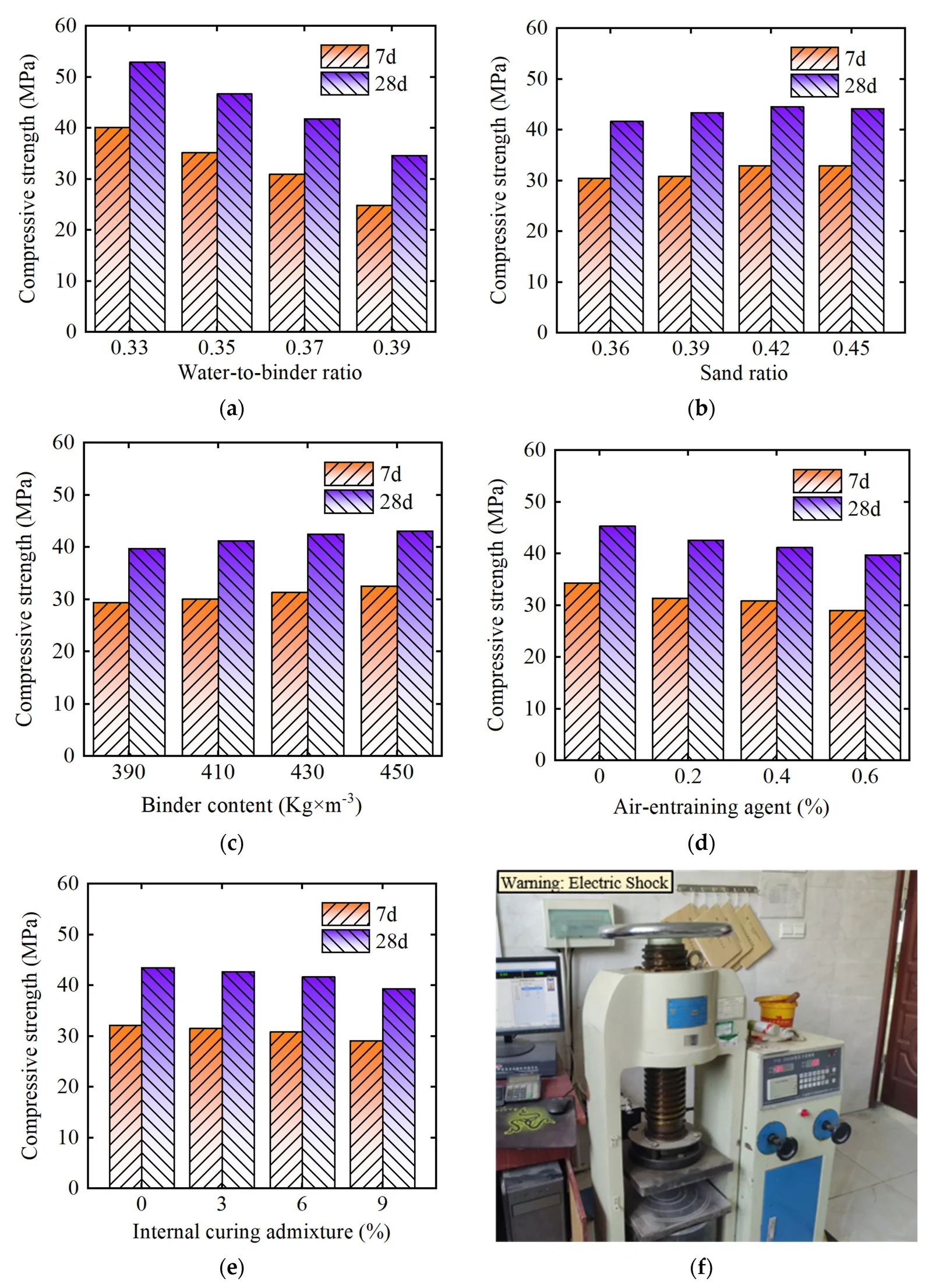
Effects of different parameters on compressive strength. (**a**) Water-to-binder ratio; (**b**) Sand ratio; (**c**) Binder content; (**d**) Air-entraining agent; (**e**) Internal curing admixture; (**f**) Photograph.

**Figure 5:**
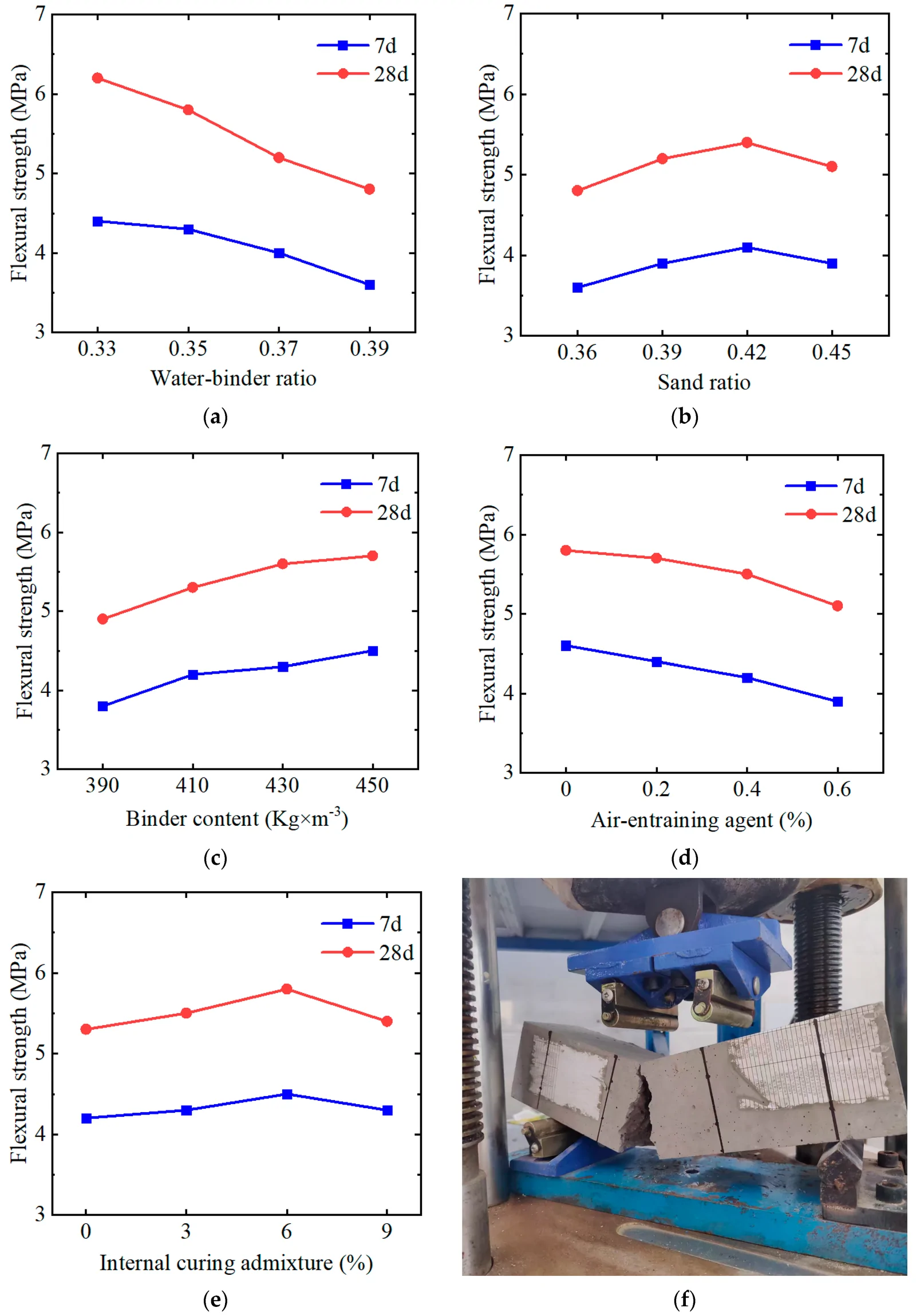
Effects of different parameters on flexural strength. (**a**) Water-to-binder ratio; (**b**) Sand ratio; (**c**) Binder content; (**d**) Air-entraining agent; (**e**) Internal curing admixture; (**f**) Photograph.

**Figure 6:**
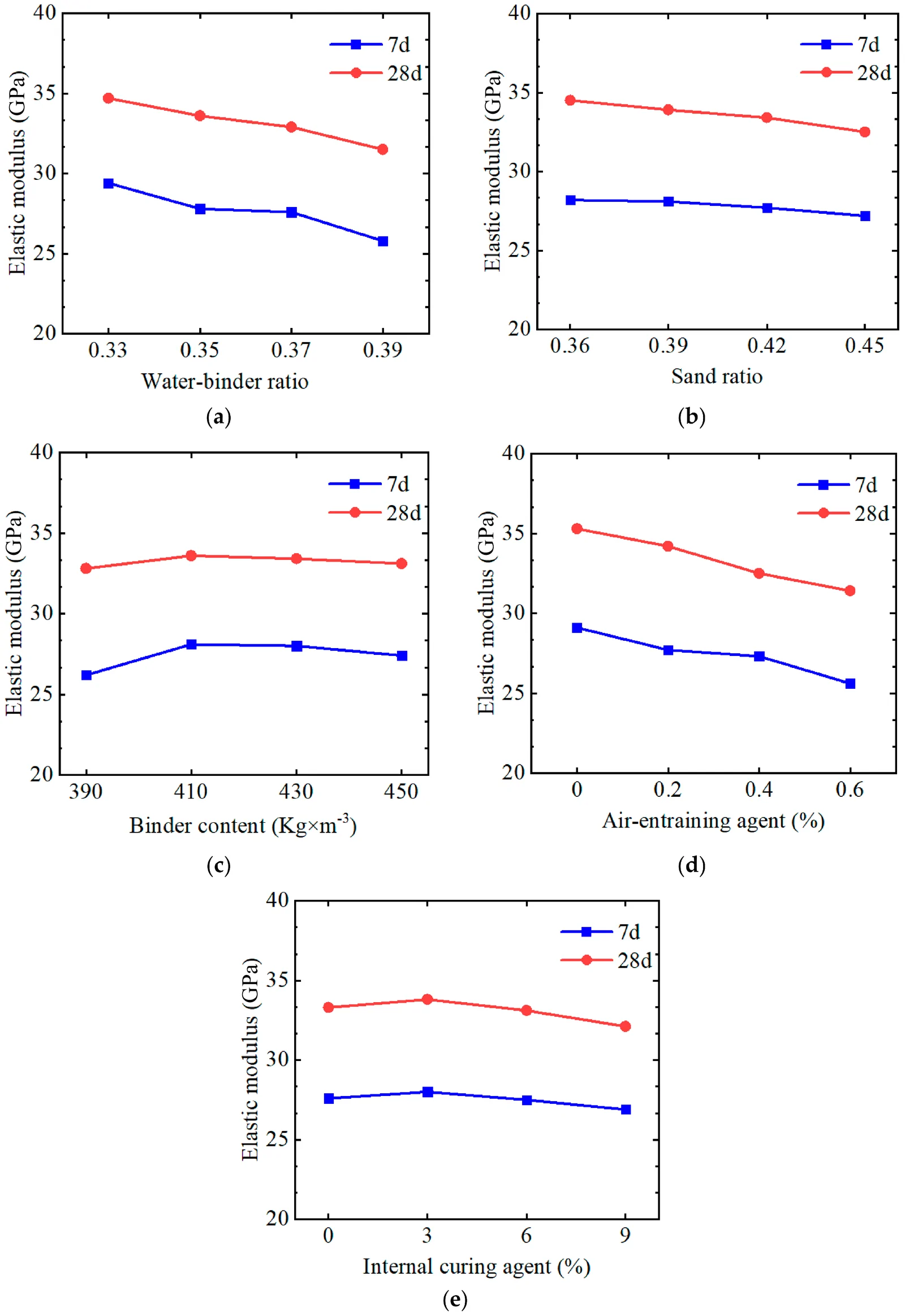
Effects of different parameters on elastic modulus. (**a**) Water-to-binder ratio; (**b**) Sand ratio; (**c**) Binder content; (**d**) Air-entraining agent; (**e**) Internal curing admixture.

The mechanical properties of concrete mixtures, including compressive strength, flexural strength, and elastic modulus, were systematically evaluated to investigate the effects of mixture parameters. These properties not only determine the load-bearing capacity of concrete but also play important roles in the cracking behavior of ballastless track slabs. The experimental results are presented in Figure 4, Figure 5 and Figure 6.

As shown in Figure 4, the compressive strength was significantly affected by the W/B, whereas the effects of sand ratio, binder content, air-entraining agent, and internal curing admixture were relatively limited. The compressive strength decreased continuously with increasing W/B, which can be attributed to the increased capillary porosity and reduced compactness of the cementitious matrix caused by excessive free water. The influence of sand ratio exhibited a relatively complex trend. Compressive strength first increases and then decreases with sand ratio, peaking at approximately 0.42. This suggests that a moderate sand ratio contributed to improved aggregate packing and a denser internal structure, while excessive sand content diminished the proportion of coarse aggregate, resulting in a slight reduction in compressive strength. The compressive strength increases with binder content from 390 to 450 kg/m^3^, owing to a greater volume of cement paste and more hydration products. The incorporation of the air-entraining agent slightly reduced compressive strength due to the introduction of additional air voids, which decreased the effective load-bearing area. Nevertheless, the reduction remained limited within the investigated dosage range. The internal curing admixture showed a minor influence on compressive strength, indicating that its primary contribution is related to deformation control rather than strength enhancement. The retained compressive strength also suggests that internal curing can be incorporated without significantly compromising mechanical performance.

As shown in Figure 5, the variation trends of flexural strength were generally consistent with those of compressive strength. However, the sensitivity to mixture parameters was more evident because flexural strength is closely associated with the integrity of the cementitious matrix and the interfacial transition zone (ITZ). Figure 5a illustrates a declining trend in flexural strength with increasing W/B, attributable to the increase in pore structure defects and the weakening of bonding between hydration products and aggregates. Figure 5b exhibits an initial increase followed by a decrease, where appropriate aggregate packing improved stress transfer efficiency, while excessive sand content reduced the restraining effect of coarse aggregates and weakened flexural resistance. Figure 5c indicates that increasing binder content improved flexural strength, and binder content had a more pronounced positive effect on flexural strength than on compressive strength. Higher binder content improves ITZ quality, which is critical for flexural performance. Figure 5d reveals that the air-entraining agent caused a slight decrease in flexural strength because entrained air voids act as stress concentration sites under bending loading. Figure 5e shows that the internal curing admixture slightly improved flexural strength, which may be attributed to the enhanced hydration degree and reduced internal defects resulting from sustained moisture supply. This result suggests that internal curing provides a favorable balance between mechanical performance and cracking resistance.

As shown in Figure 6a,d, elastic modulus decreases with increasing W/B and air-entraining agent content. This decline arises from two distinct mechanisms: a higher W/B produces a more porous cement paste with reduced stiffness, while increased air-entraining agent content introduces a greater volume fraction of compliant air voids that weaken the overall matrix rigidity. Figure 6b indicates that increasing sand ratio slightly reduces elastic modulus, because excessive sand content reduced the restraining effect of coarse aggregates. Figure 6c reveals that increasing binder content generally increased the elastic modulus owing to the formation of a denser cementitious skeleton. However, the improvement became less significant at higher binder contents, which indicates that excessive paste volume may not continuously enhance structural stiffness. Notably, the internal curing admixture showed a relatively small influence on elastic modulus, as shown in Figure 6e, indicating no substantial enhancement in mechanical stiffness.

**Figure 7:**
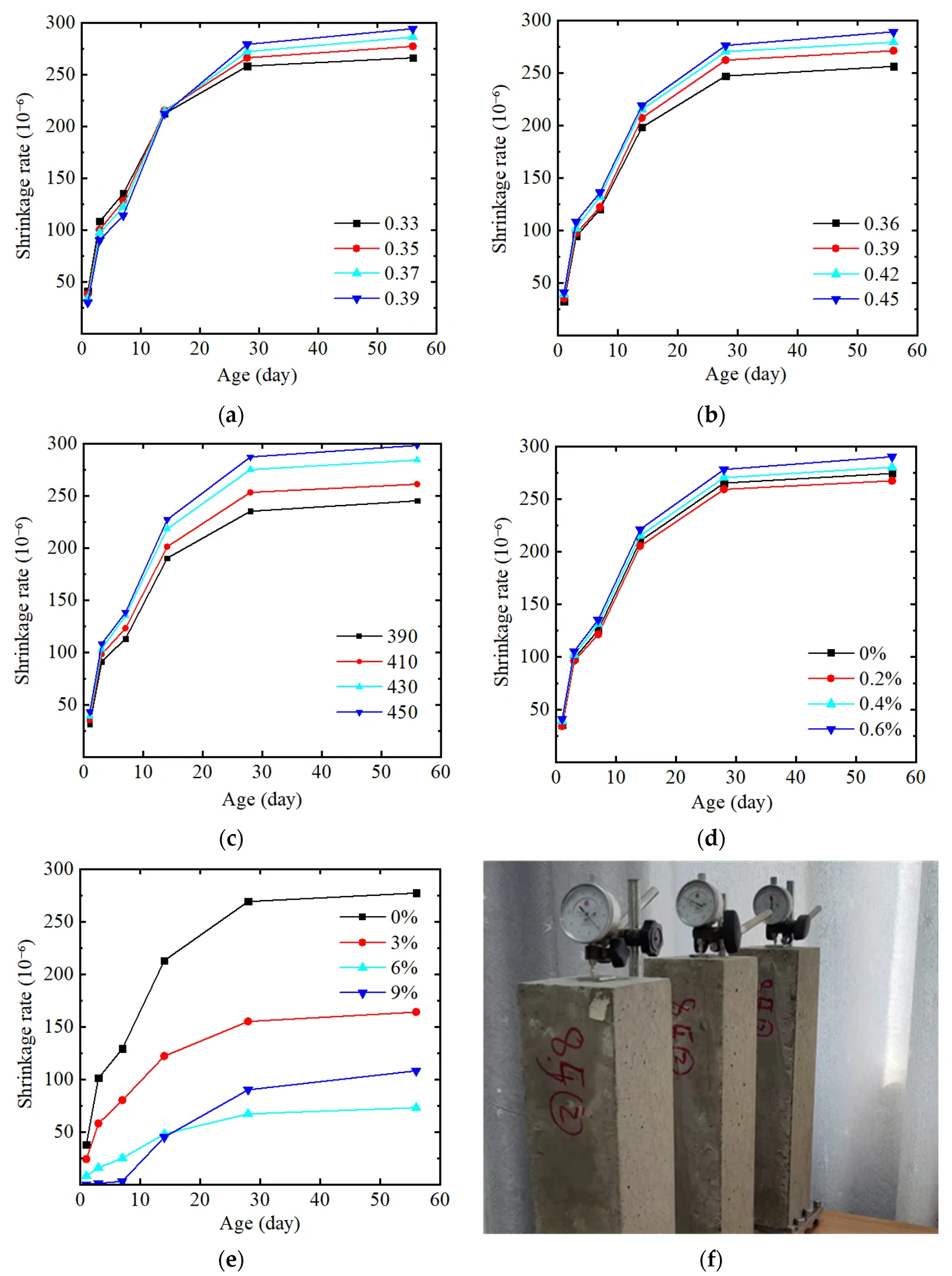
Effects of different parameters on shrinkage rate. (**a**) Water-to-binder ratio; (**b**) Sand ratio; (**c**) Binder content; (**d**) Air-entraining agent; (**e**) Internal curing admixture; (**f**) Photograph.

Shrinkage deformation is one of the primary driving factors for cracking in ballastless track slab concrete because restrained shrinkage generates tensile stress within the slab. Therefore, the influence of mixture parameters on shrinkage behavior was investigated, and the results are presented in Figure 7. The shrinkage evolution of all mixtures exhibited similar characteristics, including a rapid increase during the early stage followed by a gradually reduced growth rate. This behavior is associated with the rapid loss of moisture and intensive hydration reactions during the initial curing period. After approximately 28 d, the increase in shrinkage became relatively limited, indicating that the majority of early-age deformation had already developed.

The W/B, sand ratio, and binder content exhibited an influence on shrinkage behavior. Increasing W/B resulted in higher shrinkage deformation, which can be primarily attributed to the increased capillary pore volume and permeability of the cementitious matrix. The coarser pore structure facilitates moisture migration and evaporation during drying, thereby increasing shrinkage deformation. Increasing the sand ratio increased shrinkage deformation, which may be associated with the increased volume fraction of mortar and the reduced restraining effect of coarse aggregates. Increasing binder content significantly increased shrinkage deformation, which is mainly because a higher binder content increases the volume of cement paste, which is the primary source of shrinkage deformation in concrete.

The addition of air-entraining agent slightly increased shrinkage deformation. The introduced air voids reduce the stiffness of the concrete skeleton and may weaken the restraint effect of aggregates on the shrinking cement paste. However, the influence was relatively moderate compared with the effects of W/B and binder content.

In contrast, the internal curing admixture exhibited the most pronounced effect on reducing shrinkage deformation. The internal curing agent significantly influences the shrinkage rate development over the entire curing period, which may be attributed to the gradual release of internally stored water, which mitigates self-desiccation and maintains internal humidity during hydration. Accordingly, the incorporation of internal curing agents offers clear advantages in deformation control and anti-cracking performance. Notably, the incremental benefit from increasing the dosage from 6% to 9% appears less significant than that from 0% to 3%, implying that an optimal dosage may exist around 6-9% for practical applications. Considering the limited additional benefit at higher dosages, a cost–performance trade-off with workability constraints is recommended for practical mix design.

**Figure 8:**
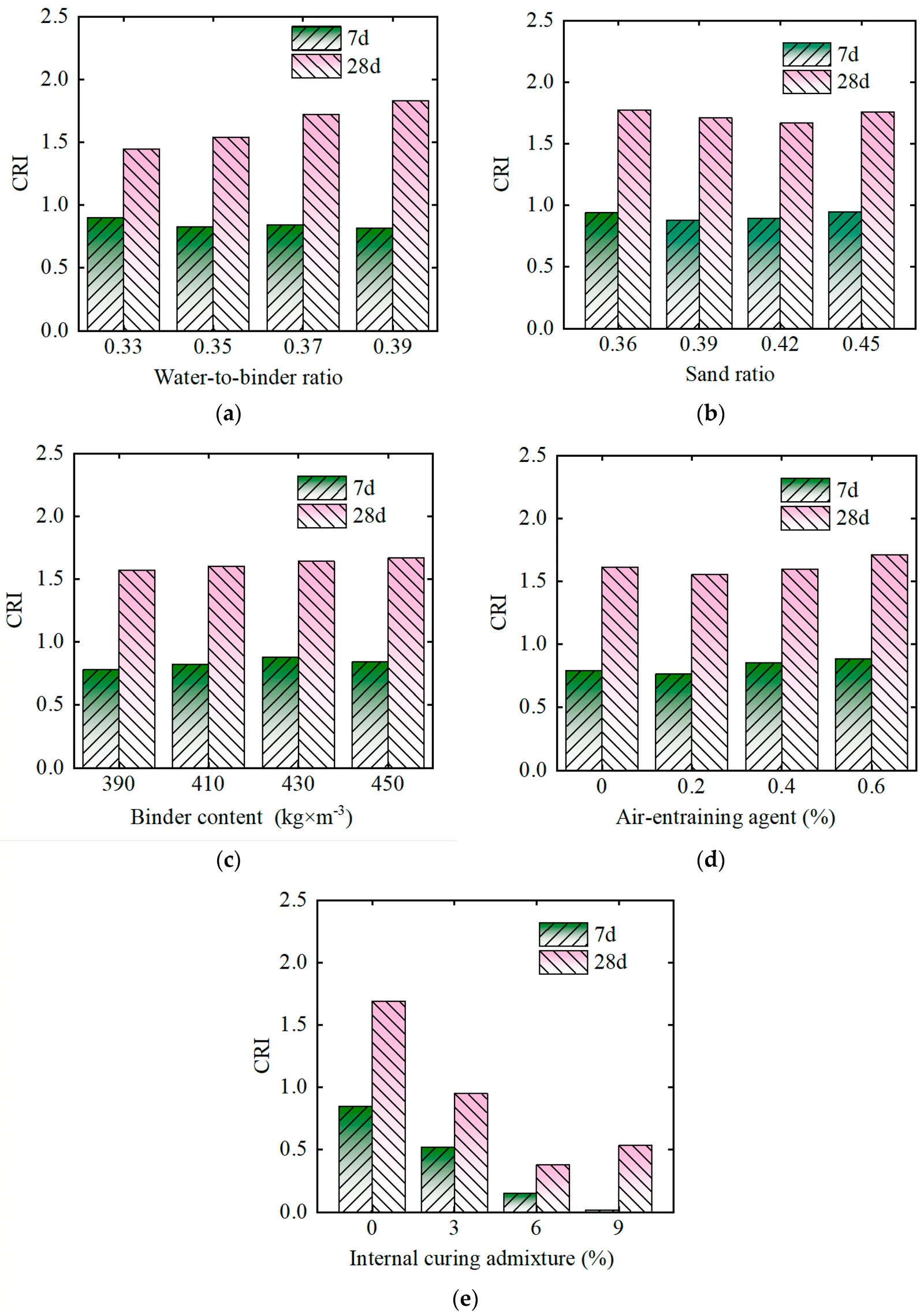
Effects of different parameters on CRI. (**a**) Water-to-binder ratio; (**b**) Sand ratio; (**c**) Binder content; (**d**) Air-entraining agent; (**e**) Internal curing admixture.

As mentioned above, the proposed CRI is a comparative index for quantitatively assessing the relative cracking resistance among different concrete mixtures. Figure 8 shows the effects of different parameters on CRI. Increasing W/B increased shrinkage deformation and reduced elastic modulus and flexural strength, leading to a deterioration in cracking resistance. Similarly, higher binder content increased CRI because the increased paste volume promoted shrinkage deformation despite improving mechanical strength. Therefore, improving strength alone cannot guarantee enhanced cracking resistance, and the deformation characteristics of concrete should be simultaneously considered. The effects of sand ratio and air-entraining agent on CRI were relatively moderate. As presented in Figure 8e, the internal curing admixture exhibited the most pronounced influence on CRI among all investigated parameters. Increasing the internal curing dosage significantly reduced the CRI value, indicating enhanced cracking resistance. This improvement may be associated with the mitigation of shrinkage deformation through gradual water release from the internal curing agent.

It should be noted that the current CRI is based on material properties measured at a specific later-age reference point and therefore does not fully describe the time-dependent evolution of cracking resistance during the early-age period of 1–7 days, when elastic modulus development, strength gain, shrinkage evolution, internal moisture variation, and stress relaxation behavior may simultaneously affect cracking resistance.

## 4. Response Surface Modeling and Mixture Optimization Based on CRI

### 4.1. Response Surface Design

The single-factor experimental results presented in Section 3 were first used to screen the five investigated mixture parameters. Among these parameters, the W/B, air-entraining agent content, and internal curing admixture content exhibited the most pronounced effects on the CRI, whereas the effects of the other two parameters were comparatively weaker within the investigated ranges. Therefore, these three parameters were selected as the key factors for the subsequent design of experiments. However, the single-factor analysis only reveals the individual influence of each mixture parameter and cannot fully capture the potential interactions among different components. A systematic optimization method considering both individual effects and interactions among mixture parameters is required.

Therefore, RSM was employed to establish the quantitative relationship between mixture parameters and CRI. By selecting representative experimental points within the design space, RSM constructs polynomial regression models and generates continuous response surfaces, which can be further used to analyze factor effects and determine optimal conditions. Compared with the central composite design (CCD), the Box–Behnken design (BBD) was adopted in this study because it requires fewer experimental runs, avoids extreme combinations of factor levels, and provides reliable estimation of quadratic regression models.

Based on the preliminary single-factor experimental results, the sand ratio was fixed at 0.42, the total binder content was maintained at 417 kg/m^3^, and the superplasticizer dosage was fixed at 1% of the binder content. Three critical mixture parameters, including water-to-binder ratio, air-entraining agent, and internal curing admixture, were selected as independent variables. The factor levels were determined as follows: water-to-binder ratios of 0.33, 0.35, and 0.37; air-entraining agent contents of 0%, 0.2%, and 0.4%; and internal curing admixture contents of 3%, 6%, and 9%. The factors and their corresponding level values in the response surface experiment are summarized in Table 8. All specimens were prepared under identical mixing and curing conditions. After casting, the molds were covered with plastic films to prevent moisture loss during initial setting. Except for shrinkage specimens, all specimens were cured in a standard curing chamber at 20 ± 2 °C and relative humidity above 95% for 28 d. The mechanical properties and shrinkage deformation were subsequently measured according to the experimental methods described previously. The obtained results were used to establish the response surface model of CRI.

### 4.2. Response Surface Results for CRI

Accordingly, a three-level BBD was employed, with W/B, air-entraining agent content, and internal curing admixture content selected as the three independent variables and the 28 d CRI as the response variable. The three independent variables and the corresponding CRI values are presented in Table 9. Based on these experimental results, a second-order polynomial regression model was developed using Design-Expert 13.0 software to describe the relationship between the selected mixture parameters and CRI:

(7) $$\begin{array}{l} Y\;=\;2.95571\;-\;5.93089A\;-\;2.24156B\;-\;0.553305C\;+\;4.82047AB+\\ 0.109333AC\;-\;0.004501BC\;+\;9.87343A^{2}\;+\;1.96275B^{2}\;+\;0.038857C^{2} \end{array}$$

where *Y* indicates the value of CRI, and the meanings of *A*, *B*, and *C* are shown in Table 8.

**Figure 9:**
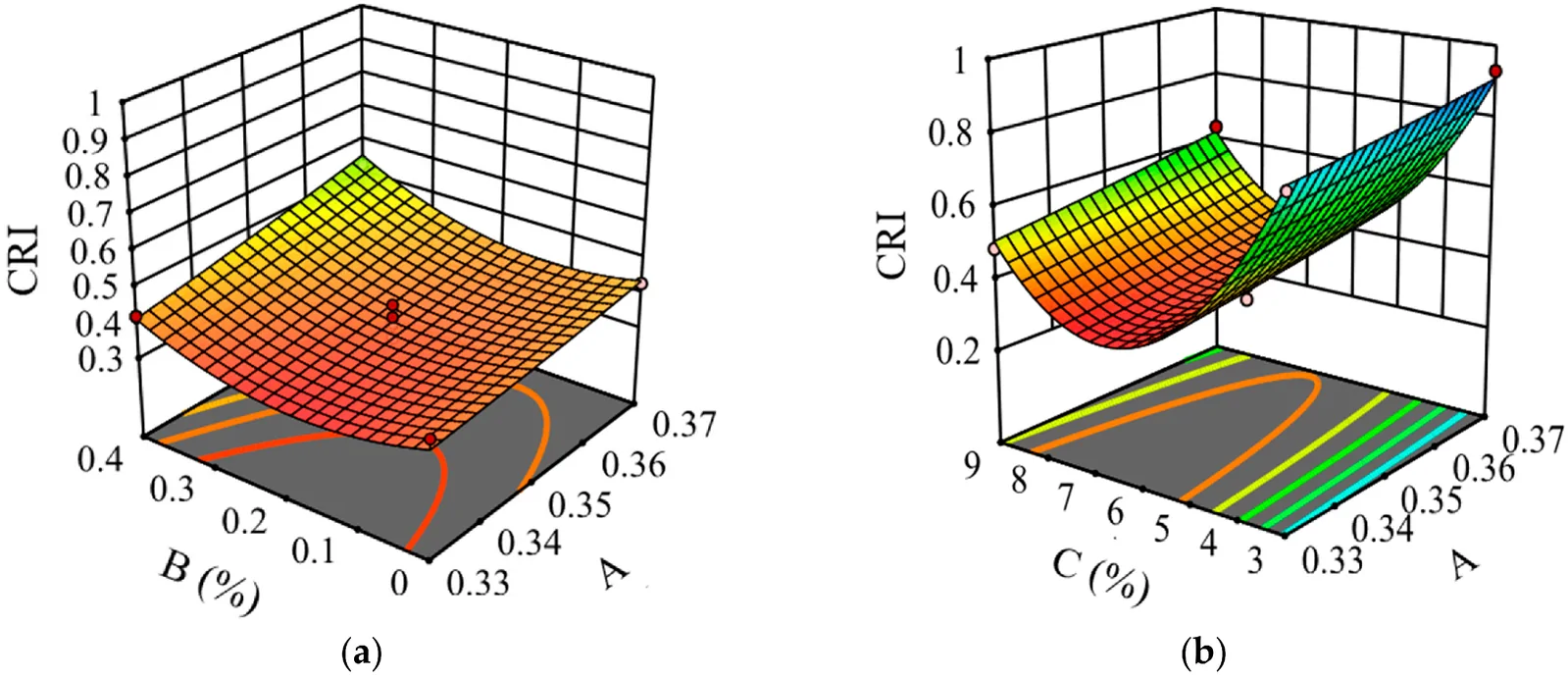

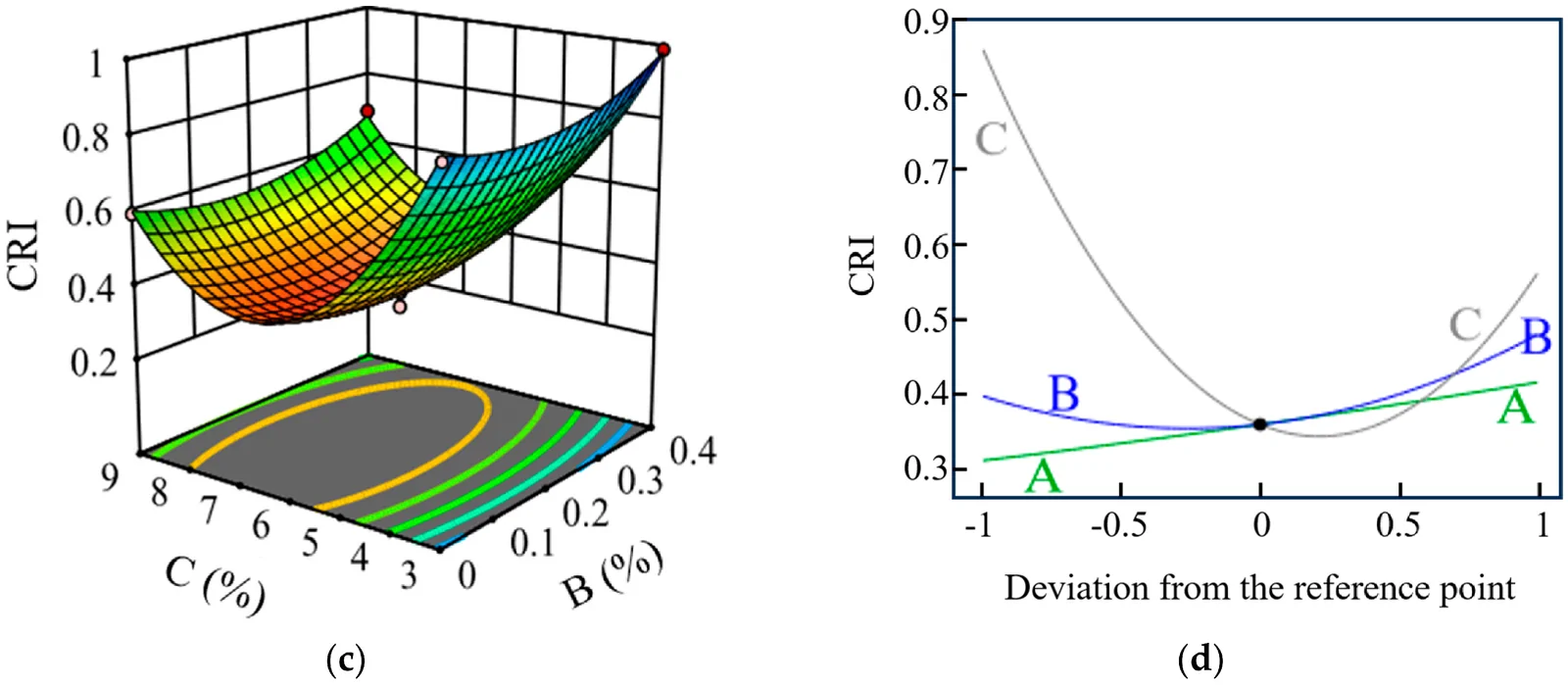
Response surface and contour plots of CRI. (**a**) Air-entraining agent and W/B; (**b**) Internal curing admixture and W/B; (**c**) Internal curing admixture and air-entraining agent; (**d**) Perturbation plot.

The response surfaces of CRI are presented in Figure 9. Specifically, Figure 9a–c present the response surfaces of CRI with respect to the three key mixture parameters. It can be clearly observed that CRI exhibits a pronounced nonlinear dependence on these parameters, with well-defined optimal regions rather than a simple monotonic trend. In addition, Figure 9d provides a perturbation plot that illustrates the sensitivity of CRI to each individual factor when deviating from the reference point. Among the three parameters, the internal curing admixture (curve C) shows the steepest variation, indicating that it is the most influential factor on cracking resistance, followed by the air-entraining agent (curve B) and W/B (curve A). These response surfaces and the perturbation plot together demonstrate that a nonlinear response model is necessary to capture the trade-off and balance among different performance indicators.

The adequacy of the response surface model was evaluated. The regression model was highly significant (F-value = 71.11, *p* < 0.0001), whereas the lack-of-fit was insignificant (F-value = 0.9858, *p* = 0.4839), indicating good agreement between the predicted and experimental results. The model achieved high fitting accuracy, with R^2^, adjusted R^2^, and predicted R^2^ values of 0.9892, 0.9753, and 0.9167, respectively. The low coefficient of variation (CV = 5.88%) and the satisfactory residual distribution further confirmed the adequacy of the model. Therefore, the developed model demonstrates preliminary validity.

### 4.3. Experimental Verification of Optimized Mixture

To obtain the optimal mixture proportions for improving the cracking resistance of ballastless track slab concrete, numerical optimization was performed using the desirability function method implemented in Design-Expert software. Since CRI represents the ratio between deformation-induced cracking demand and flexural cracking resistance, minimizing CRI was selected as the primary optimization objective. Considering the structural requirements of ballastless track slabs, the 28 d cube compressive strength was introduced as a constraint condition in the mixture optimization. According to the requirements for C40 concrete used in railway structures, the characteristic compressive strength grade is 40 MPa. This constraint ensures that the optimized mixture achieves enhanced cracking resistance while maintaining sufficient mechanical strength for structural applications.

The optimization results indicate that the optimal solution corresponds to a desirability value of 0.703. The predicted optimal mixture consists of a W/B of 0.33, an air-entraining agent content of 0.173%, and an internal curing admixture content of 6.666%. Considering experimental accuracy and practical applicability, the optimized mixture was adjusted to W/B = 0.33, air-entraining agent content = 0.17%, and internal curing admixture content = 6.67%.

Three parallel preliminary verification experiments were subsequently conducted to examine the consistency between the CRI value predicted by the RSM and the experimentally measured CRI value of the optimized mixture. The experimental results of the optimized mixture were compared with the predicted values obtained from the response surface model. As summarized in Table 10, the relative error between the measured mean value of the three replicates and the predicted CRI value is 3.81%. This preliminary verification primarily demonstrates the predictive consistency of the RSM and the agreement between the optimized mixture performance and the predicted CRI value. It should be noted that the three replicate specimens do not constitute independent validation of CRI as a direct measure of actual cracking resistance. Within the scope of comparative evaluation, the optimized mixture proportion provides a feasible solution for balancing mechanical performance and the comparative cracking resistance characteristics represented by CRI in ballastless track slab concrete mixtures.

It should be noted that this verification is based on only three specimens and therefore represents a preliminary experimental consistency check of the optimization framework rather than a comprehensive validation of the proposed method. Further experimental data with larger sample sizes are required to more rigorously assess the robustness and broader applicability of the proposed optimization framework.

## 5. Conclusions

This study proposed a cracking resistance evaluation and optimization framework for ballastless track slab concrete by establishing a CRI and integrating it with response surface methodology. Based on the experimental investigation and response surface optimization, the following conclusions can be drawn:

(1) The cracking behavior of ballastless track slab concrete is essentially governed by the competition between deformation-induced stress demand and material resistance capacity. Based on this concept, the proposed CRI integrates shrinkage deformation, elastic modulus, and flexural strength into a unified comparative mixture-level evaluation framework. Compared with conventional single-parameter indicators, CRI simultaneously considers the adverse effect of deformation-induced stress development and the beneficial contribution of flexural resistance.

(2) The proposed CRI captures the combined effects of the investigated mixture parameters on the comparative cracking resistance within the experimental dataset. Increasing the water-to-binder ratio increased shrinkage deformation while reducing mechanical resistance, resulting in an increased CRI value and deteriorated cracking resistance. Binder content exhibited a dual effect by improving mechanical properties while increasing shrinkage-induced cracking potential. These results demonstrate that enhancing strength alone cannot guarantee improved cracking resistance, and the balance between deformation control and resistance capacity should be considered.

(3) The internal curing admixture was identified as an effective approach for reducing CRI values within the investigated mixtures. Increasing the dosage of TK-ICM reduced shrinkage deformation and CRI, which may be attributed to the internal moisture regulation effect of internal curing and the potential mitigation of self-desiccation during hydration. This finding highlights that controlling deformation-induced stress development is an important strategy for improving the cracking resistance of ballastless track slab concrete.

(4) An RSM with CRI as the optimization objective was established, based on which an optimized mixture proportion satisfying the strength requirement of C40 concrete and achieving a lower CRI value was obtained. Three parallel experiments were conducted for the optimized mixture, and the relative error between the measured mean CRI value and the RSM-predicted value was only 3.81%, indicating that the developed RSM provides good predictive consistency and can serve as an effective tool for mixture optimization.

The present results do not constitute independent evidence for the universality of CRI as a direct cracking criterion for structural members. The applicability of CRI requires further validation through recognized restrained cracking tests. Moreover, the current framework does not explicitly account for early-age behavior, creep, stress relaxation, fracture behavior, and actual environmental conditions; thus, the practical applicability of CRI beyond mixture-level comparative evaluation requires further verification.

## Figures and Tables

**Table 1:** Performance test results of the internal curing admixture.

Test Item		Specified Requirement	Test Result
Chloride ion content (%)		≤0.06%	0.041%
Alkali content (%)		≤0.8%	0.43%
SO_3_ content (%)		/	18.29%
Setting time difference	Initial setting	−90 min~+120 min	−20 min
	Final setting		−12 min
Compressive strength ratio	3 d	≥80%	83%
	28 d	≥90%	95%
Shrinkage ratio	12 h	≤60%	48%
	28 d	≤80%	73%
Cracking resistance	28 d	No cracking	No cracking

**Table 2:** Concrete mixture proportions under different W/Bs.

Group	Raw Material Content (k g × m^−3^)						W/B
	Cement	Fly Ash	Sand	Coarse Aggregate	Superplasticizer	Water	
W1	292	125	773	1068	4.17	138	0.33
W2	292	125	770	1063	4.17	146	0.35
W3	292	125	766	1059	4.17	154	0.37
W4	292	125	763	1053	4.17	163	0.39

**Table 3:** Concrete mixture proportions under different sand ratios.

Group	Raw Material Content (kg × m^−3^)						Sand Ratio
	Cement	Fly Ash	Sand	Coarse Aggregate	Superplasticizer	Water	
S1	292	125	658	1171	4.17	150	0.36
S2	292	125	713	1116	4.17	150	0.39
S3	292	125	768	1061	4.17	150	0.42
S4	292	125	823	1006	4.17	150	0.45

**Table 4:** Concrete mixture proportions under different binder content values.

Group	Raw Material Content (kg × m^−3^)						Binder Content
	Cement	Fly Ash	Sand	Coarse Aggregate	Superplasticizer	Water	
B1	273	117	784	1082	3.9	140	390
B2	287	123	772	1066	4.1	148	410
B3	301	129	761	1050	4.3	155	430
B4	315	135	749	1035	4.5	162	450

**Table 5:** Concrete mixture proportions under different air-entraining agent dosages.

Group	Raw Material Content (kg × m^−3^)						Air-Entraining Agent (%)
	Cement	Fly Ash	Sand	Coarse Aggregate	Superplasticizer	Water	
A1	292	125	768	1061	4.17	150	0
A2	292	125	768	1061	4.17	150	0.2
A3	292	125	768	1061	4.17	150	0.4
A4	292	125	768	1061	4.17	150	0.6

**Table 6:** Concrete mixture proportions under different internal curing admixture dosages.

Group	Raw Material Content (kg × m^−3^)						Internal Curing Admixture (%)
	Cement	Fly Ash	Sand	Coarse Aggregate	Superplasticizer	Water	
I1	292	125	768	1061	4.17	150	0
I2	279	125	768	1061	4.17	150	3
I3	267	125	768	1061	4.17	150	6
I4	254	125	768	1061	4.17	150	9

**Table 7:** Experimental results for workability.

Group	Slump (mm)	Air Content (%)	Group	Slump (mm)	Air Content (%)
W1	155	4.9	S1	165	5.1
W2	170	5.3	S2	175	5.1
W3	180	5.6	S3	180	5.3
W4	210	5.7	S4	175	5.6
B1	170	6.3	A1	155	3.1
B2	175	6	A2	175	4.8
B3	185	5.6	A3	180	5.8
B4	190	5.4	A4	185	6.3
I1	210	5.1	—	—	—
I2	195	5.4	—	—	—
I3	175	5.8	—	—	—
I4	150	6.3	—	—	—

**Table 8:** The factors and their corresponding level values.

Factors	Level Values		
	−1	0	1
W/B (A)	0.33	0.35	0.37
Air-entraining agent (B)	0	0.2	0.4
Internal curing admixture (C)	3	6	9

**Table 9:** Results of three-level input and output variables.

Number	A	B (%)	C (%)	CRI
1	0.33	0	6	0.40
2	0.37	0	6	0.43
3	0.33	0.4	6	0.42
4	0.37	0.4	6	0.53
5	0.33	0.2	3	0.80
6	0.37	0.2	3	0.93
7	0.33	0.2	9	0.49
8	0.37	0.2	9	0.64
9	0.35	0	3	0.88
10	0.35	0.4	3	0.99
11	0.35	0	9	0.59
12	0.35	0.4	9	0.69
13	0.35	0.2	6	0.41
14	0.35	0.2	6	0.31
15	0.35	0.2	6	0.37
16	0.35	0.2	6	0.35
17	0.35	0.2	6	0.36

**Table 10:** Comparison of predicted and experimental CRI values.

Indicator	Predicted Value	Measured Value (Replicate 1)	Measured Value (Replicate 2)	Measured Value (Replicate 3)	Measured Mean
CRI	0.2995	0.3076	0.3200	0.3052	0.3109

## Data Availability

The original contributions presented in this study are included in the article. Further inquiries can be directed to the corresponding author.
